# Data from proteomic analysis of bovine Longissimus dorsi muscle associated with intramuscular fat content

**DOI:** 10.1016/j.dib.2018.06.004

**Published:** 2018-06-09

**Authors:** Mirele D. Poleti, Luciana C.A. Regitano, Gustavo H.M.F. Souza, Aline S.M. Cesar, Rosineide C. Simas, Bárbara Silva-Vignato, Gabriella B. Oliveira, Sónia C.S. Andrade, Luiz C. Cameron, Luiz L. Coutinho

**Affiliations:** a Department of Animal Science, “Luiz de Queiroz” College of Agriculture, University of São Paulo/ESALQ, Piracicaba, SP 13418-900, Brazil; b Embrapa Pecuária Sudeste, São Carlos, SP 13560-970, Brazil; c MS Applications and Development Laboratory Waters Corporation, São Paulo, SP 06455-020, Brazil; d Department of Animal Science, College of Animal Science and Food Engineering, University of São Paulo/FZEA, Pirassununga, SP 13635-900, Brazil; e Laboratory of Protein Biochemistry - Federal University of State of Rio de Janeiro/UNIRIO, Rio de Janeiro, RJ 22290-255, Brazil; f Department of Biochemistry and Sportomics - Olympic Laboratory - Brazil Olympic Committee, Rio de Janeiro, RJ, Brazil

## Abstract

The proteomic data presented in this article are associated with the research article entitled “*Longissimus dorsi* muscle label-free quantitative proteomic reveals biological mechanisms associated with intramuscular fat deposition” published in Journal of Proteomics [1]. In this article, we characterized the proteomic profile of bovine *Longissimus dorsi* muscle from Nelore steers and identified differentially abundant proteins associated with the intramuscular fat (IMF) content. An integrated transcriptome-assisted label-free quantitative proteomic approach by High Definition Mass Spectrometry (HDMS^E^) was employed to identify and quantify the proteins. A functional enrichment analysis using the differentially abundant proteins list was performed to understand the biological processes involved in IMF deposition. Moreover, to explore and clarify the biological mechanisms that influence IMF content, the mRNA data for the same trait from Cesar and collaborators [2] obtained by RNA-sequencing technology was compared with proteomic data. The mRNA data is deposited in the European Nucleotide Archive (ENA) repository (EMBL-EBI), under accession PRJEB13188.

**Specifications Table**

**Table:** 

Subject area	*Agriculture, Biology*
More specific subject area	*Label-free quantitative proteomics*
Type of data	*Tables*
How data was acquired	*MS/MS data acquired from Synapt G2-S HDMS mass spectrometer (Waters) combined with nanoACQUITY UPLC 2D Technology system (Waters).*
	*mRNA data from RNA-sequencing technology.*
Data format	*Filtered and analyzed.*
Experimental factors	*No sample pretreatment applied*
Experimental features	*Analysis of protein profile and gene expression from bovine Longissimus dorsi muscle.*
Data source location	*“Luiz de Queiroz” College of Agriculture, University of São Paulo, Piracicaba, São Paulo, Brazil.*
Data accessibility	*Proteomic data are presented in this article. The mRNA data is available in the European Nucleotide Archive (ENA) repository (EMBL-EBI), under accession PRJEB13188. [*http://www.ebi.ac.uk/ena/data/view/PRJEB13188*]*

**Value of the data**

• A customized protein database from RNA sequencing data of the Nelore (*Bos indicus*) *Longissimus dorsi* muscle promoted a better characterization of the protein profile of the samples.
• Label-free quantitative proteomic approach by High Definition Mass Spectrometry is useful to explore the biological mechanisms involved in differences of the intramuscular fat content.
• Integrative analysis of the proteomic and mRNA dataset is an exciting strategy to understand the physiological phenomena more accurately.

## 1 Data

Intramuscular fat (IMF), or marbling, is a relevant trait for the sensory attributes and nutritional values of beef. Individual differences of IMF content are notable and affect production systems. In this way, understanding of the biological mechanisms that regulate IMF deposition in beef cattle is essential to improve beef quality. The proteomic profile of *Longissimus dorsi* muscle of a group of 20 Nelore male cattle with extreme genomic estimated breeding values (GEBV) for intramuscular fat (IMF) content was compared [1]. A Nelore protein database was built from RNA-sequencing data from LD muscle for protein identification and quantification. Functional enrichment analysis was performed using the list of the differentially abundant proteins (DAPs) between groups, and the list of differentially expressed genes (DEGs) obtained from Cesar and collaborators [2] to better explore the biological mechanisms that influence intramuscular fat deposition.

## 2 Experimental design, materials and methods

*Longissimus dorsi* muscle samples from Nelore cattle at 24±1 month of age were collected during the first-hour *post-mortem*. We selected 10 samples with higher (H) and 10 samples with lower (L) genomic estimated breeding values (GEBV) for intramuscular fat to carried out proteomic analysis (Table S1).The IMF content (%), backfat thickness (BFT, mm) and ribeye area (REA, cm2) were determined as described in [3], [4]. The total proteins were extracted, desalted, denatured, reduced, alkylated and enzymatically digested with trypsin.

## 3 Proteome analysis

The peptides samples were separated using nanoACQUITY UPLC 2D Technology system [5] and identified by Synapt G2-S High Definition mass spectrometer (Waters, Manchester, UK). A nanoACQUITY UPLC HSS T3 1.8 µm, 75 µm×15 cm column (pH 3) was used in conjunction with a reverse phase (RP) XBridge BEH130 C18 5 µm 300 µm×50 mm nanoflow column (pH 10). Typical on-column sample loads were 500 ng of total protein digests for each of the 3 fractions (500 ng/fraction/load). Data were acquired under multiplexed data-independent (DIA) scanning with added specificity and selectivity of a non-linear ‘T-wave’ ion mobility (HDMS^E^) device. The MS spectra were acquired with Waters MassLynx v.4.1 software and processed using Progenesis QI for Proteomics (QIP) 2.0 software. For protein identification and quantification, the raw data were searched against a Nelore transcriptome database built from RNA-sequencing data from LD muscle. Proteins identified are shown in Table S2. Label-free protein quantification values were generated based on the label-free Hi3 method [6]. Only proteins present in at least eight biological replicates and identified with at least two peptides were used in differential abundance analysis (Table S3). Differentially abundant proteins (DAP) were considered at *p*-value <0.05 by ANOVA test from Progenesis software (Table S4).

## 4 Functional enrichment analysis

The list of the differentially abundant proteins (*p*<0.05) between groups [1] was submitted to functional enrichment analysis by Biological Network Gene Ontology – BiNGO [7], an app from Cytoscape [8]. The REVIGO tool [9] was utilized to summarize the redundant GO terms generated (Table S5). Networks analysis were carried out by Ingenuity Pathway Analysis software (Table S6). Afterward, we compared the proteomics data obtained in this study and the RNA-sequencing data collected from a previous study of our group performed for the same biological samples [2] to had better understand of the biological mechanisms involved in IMF content. The differentially abundant proteins and differentially expressed genes lists were analyzed separately and later combined using DAVID (Database for Annotation, Visualization, and Integrated Discovery) version 6.7 [10]. The common terms enriched for both lists of DEGs and DAPs are shown in Table S7. The terms enriched for DEGs and DAPs lists analyzed together are shown in Table S8, and the terms enriched for DEGs, and DAPs lists analyzed separately are shown in Tables S9 and S10, respectively.
